# An Unusual Case of Coarctation of the Aorta Repair: A 35-Year Follow-Up

**DOI:** 10.7759/cureus.95987

**Published:** 2025-11-03

**Authors:** Hugh Jacobs, Gianni D Angelini

**Affiliations:** 1 Cardiac Surgery, Bristol Heart Institute, Bristol University, Bristol, GBR

**Keywords:** aberrant subclavian artery, adult congenital heart disease, bypass graft, coarctation of the aorta, long-term follow-up

## Abstract

Coarctation of the aorta (CoA) is a congenital narrowing of the thoracic aorta, most often diagnosed in childhood. In adults, surgical repair is less common, with endovascular stenting now preferred when feasible. Open repair, however, remains essential in anatomically complex cases. We describe a male patient who presented in 1988 at age 34, with arterial hypertension and right arm hypoplasia. Imaging revealed severe post-ductal coarctation and an anomalous right subclavian artery arising immediately distal to the narrowed segment. During surgery, trial cross-clamping of the coarctation and aberrant artery resulted in a critical drop in distal pressure, precluding conventional resection and interposition grafting. A bypass repair with a 20 mm Dacron tube graft was performed. The postoperative course was uncomplicated. Thirty-five years later, computed tomography (CT) imaging confirmed the continued competence of the graft. This case illustrates the importance of tailoring CoA repair to individual anatomy. While interposition grafting remains the standard open technique in adults, bypass grafting provides a safe and durable alternative when cross-clamping is not tolerated or anomalous anatomy is present. Long-term follow-up demonstrates excellent durability of bypass grafting, reinforcing its continued role in the surgical armamentarium.

## Introduction

Coarctation of the aorta (CoA) is a congenital narrowing of the thoracic aorta, typically at the juxtaductal position near the ligamentum arteriosum, and accounts for 4%-8% of congenital heart disease [1,2]. Although usually diagnosed in infancy or childhood, milder forms may present in adulthood, often during evaluation for systemic hypertension. Associated cardiovascular anomalies are common, particularly bicuspid aortic valve (BAV) and intracranial aneurysms, while extracardiac vascular anomalies such as an aberrant right subclavian artery occur in up to 5% of patients [1,2]. Untreated CoA carries a poor prognosis, with a mean survival of only 35 years, most commonly due to heart failure, aortic rupture, or cerebrovascular events [3].

Contemporary data emphasize that the management of adult coarctation requires individualized assessment based on anatomical and hemodynamic parameters. Current European Society of Cardiology (ESC) guidelines recommend intervention in hypertensive patients with an invasive peak-to-peak gradient ≥ 20 mmHg, with stenting preferred in anatomically suitable adults [4]. Similarly, the 2018 American Heart Association (AHA)/American College of Cardiology (ACC) guidelines recommend intervention for patients with a peak-to-peak gradient ≥ 20 mmHg or with imaging evidence of significant collateral flow, hypertension, or left ventricular hypertrophy, even in the absence of a severe gradient [5]. Recent work by Egbe et al. demonstrated that the aortic isthmus ratio has the strongest correlation with the left ventricular mass index, serving as a reliable anatomical marker of disease severity [6]. Furthermore, Agasthi et al. and Velayudhan and Idhrees have underscored that adult CoA is a generalized arteriopathy requiring lifelong surveillance, often involving multimodality imaging and multidisciplinary management [7,8].

Open repair remains indispensable in patients with complex anatomy or unfavorable vascular morphology. In adults, surgical options include resection with interposition grafting, the standard open repair, and extra-anatomic bypass grafting, typically reserved for anatomically challenging or reoperative cases [9,10].

We present the case of an adult patient with post-ductal CoA and an anomalous right subclavian artery, in whom conventional interposition grafting was precluded intraoperatively. A bypass Dacron tube graft was performed in 1989, and long-term follow-up 35 years later demonstrates continued graft competence and excellent outcome.

## Case presentation

**Figure 1:**
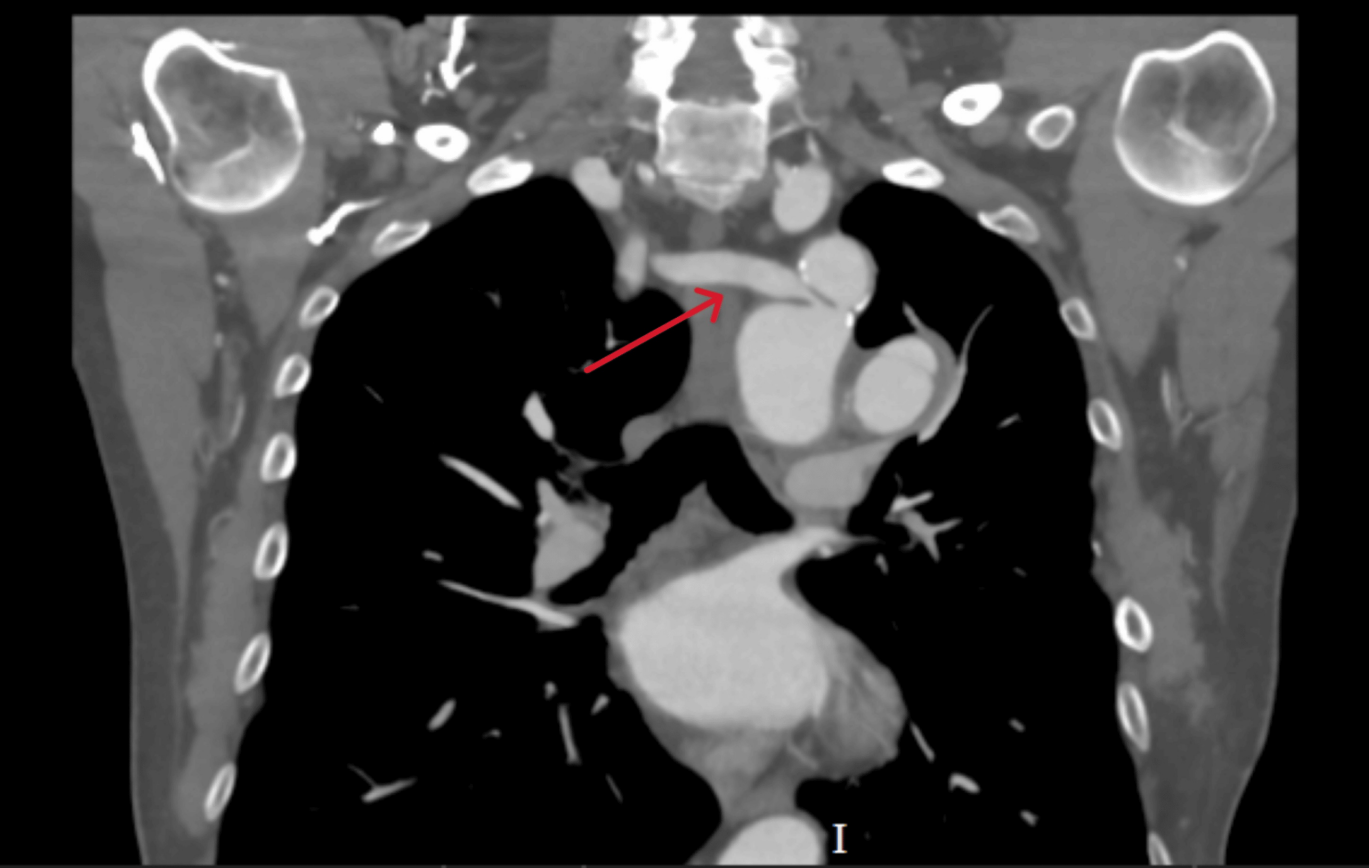
Coronal CT image showing an anomalous right subclavian artery (red arrow) arising from the aorta, distal to coarctation. CT: computed tomography

In 1988, a 34-year-old man from Italy, with no prior medical history and otherwise fit and healthy, presented to his general practitioner with persistently elevated blood pressure (170-180/90-95 mmHg). He was asymptomatic, with no history of chest pain, dyspnea, syncope, or exercise intolerance. On examination, the only abnormal finding was an asymmetry between the upper limbs, with the right arm noticeably smaller and less developed than the left. This prompted further investigations, which revealed a severe CoA in the typical post-ductal position. Imaging also demonstrated an anomalous right subclavian artery arising from the posteromedial aspect of the aorta, immediately distal to the coarctation (Figure 1). Given these findings, surgical repair was recommended.

The patient underwent surgery at University Hospital Cardiff in Wales the following year. Intraoperatively, access was established via a left posterolateral thoracotomy through the bed of the excised fifth rib. The coarctation was located in the usual position for post-ductal coarctation, and the ductus arteriosus was found to be completely obliterated. Except for the first intercostal artery, the left-sided intercostal arteries were not grossly enlarged and originated from the lateral aspect of the aorta rather than the posterior aspect. The anomalous right subclavian artery arose from the posteromedial aspect of the aorta, immediately distal to the coarctation. A trial cross-clamp of the coarctation segment and the anomalous right subclavian artery produced an unacceptable drop in distal aortic pressure, making conventional resection unfeasible without a left ventricle-to-distal aortic shunt.

Consequently, the surgical team decided to bypass the coarctation using a 20 mm Dacron tube graft. The mediastinal pleura was opened longitudinally over the aorta and retracted with stay sutures. All major vessels were dissected free and taped, and the ligamentum arteriosum was divided between ligatures. The first left intercostal artery was also divided between ligatures. Side-biting clamps were applied to the proximal and distal aorta, and the graft was sutured in place using continuous 4/0 Prolene (Ethicon, Inc., Somerville, US) for each anastomosis. The graft diameter closely matched that of the proximal aorta, which was smaller than the distal aorta, as commonly seen in coarctation. Throughout the procedure, the distal aortic pressure remained stable, with a mean of 65-75 mmHg. The chest was then closed in the usual manner.

**Figure 2:**
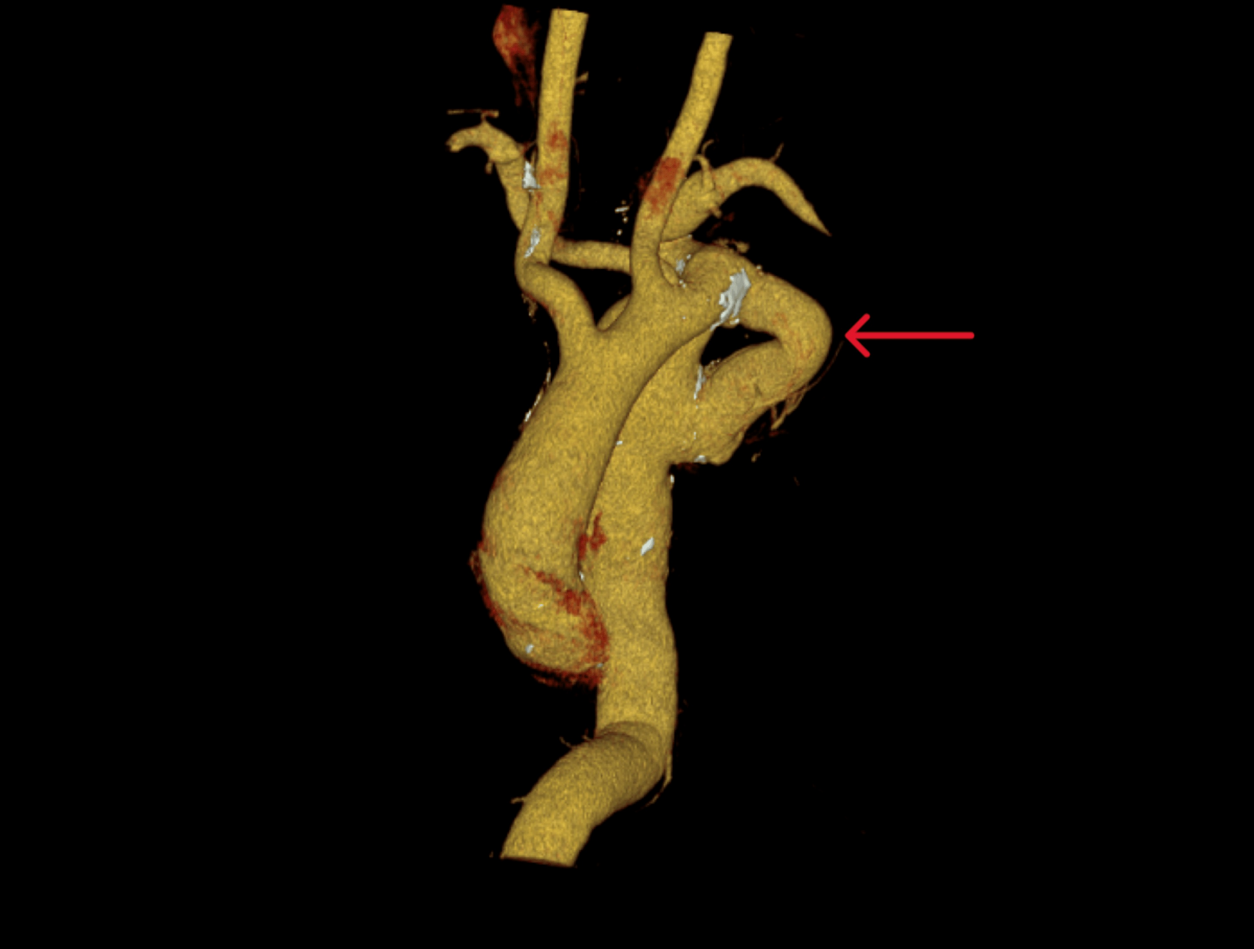
3d reconstruction of extra-anatomic bypass graft (red arrow).

**Figure 3:**
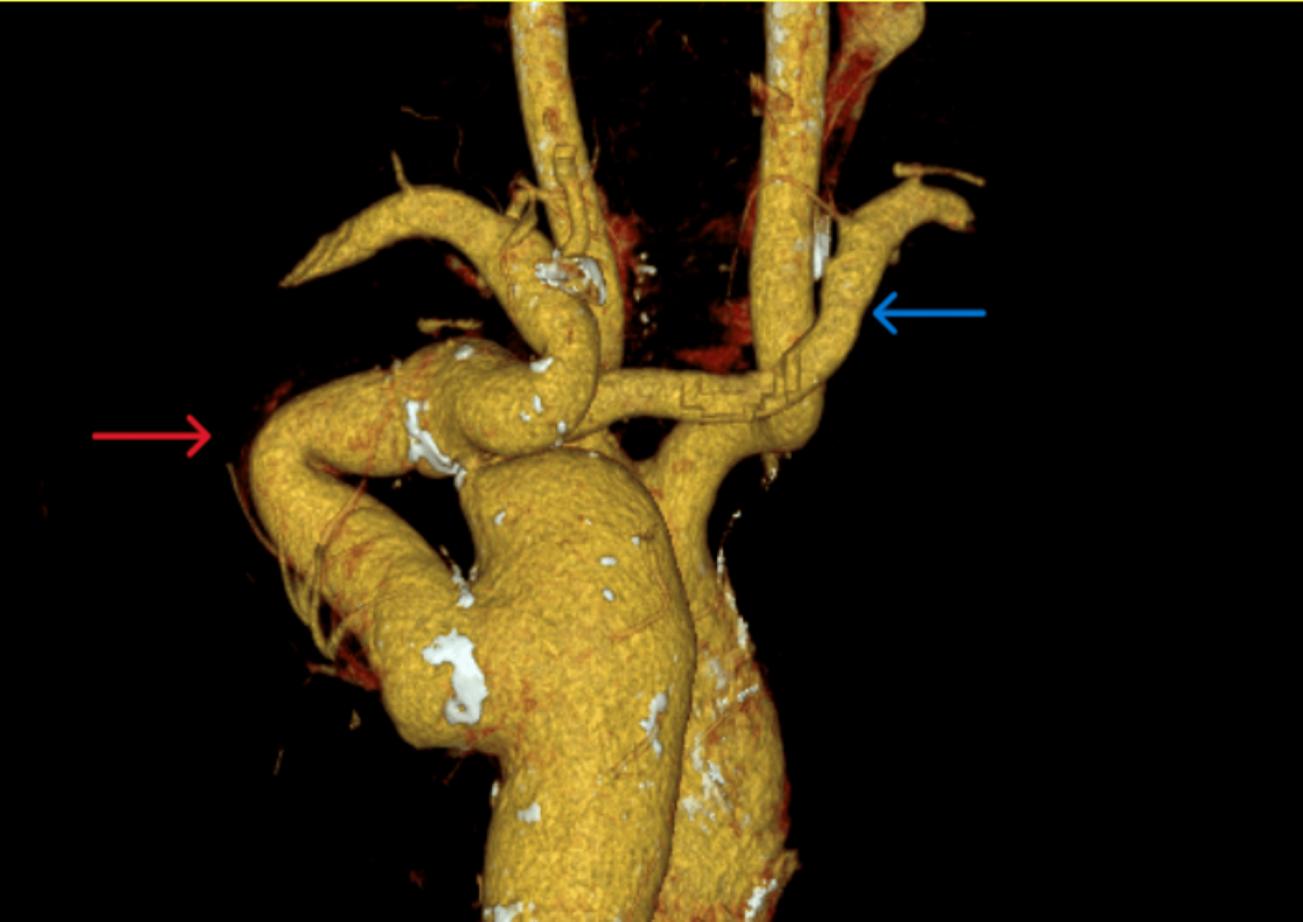
3d reconstruction of extra-anatomic bypass graft (red arrow); an aberrant right subclavian artery (blue arrow) can be seen arising from the posteromedial aspect of the aorta, distal to the coarctation.

The patient had a good postoperative outcome and was discharged with no complications. His graft was competent, and he was asymptomatic. Below, we provide the images from a computed tomography (CT) scan that was done 35 years later in 2025 (Figures 2, 3).

## Discussion

CoA is a congenital narrowing of the thoracic aorta, most often occurring in the region of the ductus arteriosus at the aortic isthmus. It represents 4%-8% of congenital heart disease and occurs in approximately one in 2,500 live births, with a male predominance [1,2]. Several embryologic theories have been proposed, including Edwards’ hypothetical double aortic arch model, reduced intrauterine blood flow with underdevelopment of the arch, and aberrant incorporation of ductal tissue into the aortic wall [2].

Typically, the CoA is located in the juxtaductal position; however, ectopic sites, such as the ascending, descending thoracic, or abdominal aorta, are rare [1,2]. CoA is frequently associated with other congenital lesions, including the BAV in up to 85% of cases, ascending aortic aneurysm, subaortic or supravalvular aortic stenosis, Shone complex, or mitral valve abnormalities [1,2]. Syndromic associations include Turner and Williams-Beuren syndromes, while extracardiac vascular anomalies such as an aberrant right subclavian artery occur in approximately 4%-5% of cases [1]. This was the case in our patient, who had this rare aberrant right subclavian artery. Intracranial aneurysms are also found to be present in up to 10% of patients [1,2].

The clinical presentation depends on severity. Severe forms usually present in infancy, whereas milder lesions may remain undetected until adulthood, often discovered during the evaluation of arterial hypertension [1,2]. Without repair, natural history is poor, with a median survival of 35 years and 75% mortality by mid-40s due to heart failure, aortic rupture/dissection, or intracranial hemorrhage [3]. Current ESC guidelines recommend repair (surgical or catheter-based) in hypertensive patients with a confirmed invasive peak-to-peak gradient ≥ 20 mmHg, with catheter-based stenting preferred in suitable anatomies (Class I, Level C) [4].

The operative management of CoA has evolved considerably. In children, standard surgical techniques include resection with end-to-end or extended end-to-end anastomosis, prosthetic patch aortoplasty, and subclavian flap repair [9]. Resection with end-to-end or extended end-to-end anastomosis is the most commonly performed operation in the pediatric population, as it restores continuity of the native aorta and allows for somatic growth. Subclavian flap aortoplasty and prosthetic patch aortoplasty have also been employed but are associated with a higher risk of late aneurysm formation and are, therefore, less commonly used today [9].

In adults, however, the aortic wall is less compliant, collaterals are more developed, and extensive calcification may be present, making these techniques technically difficult and less applicable. Resection of the stenotic segment followed by interposition of a prosthetic graft has become the reference open technique in adults, offering excellent long-term durability, with survival beyond 90% at 20 years [10]. However, this approach requires cross-clamping of the diseased segment and may be technically hazardous in the presence of complex collaterals or anomalous branch vessels. Extra-anatomic bypass grafts, such as descending aortic bypasses or ascending-to-descending aorta conduits, are especially useful in redo operations or when standard repair is unsafe, and allow correction without interrupting flow through the narrowed segment [9]. More recently, endovascular stenting has become the first-line treatment in suitable anatomies, due to its less invasive nature and low perioperative morbidity, though the long-term durability of stents requires ongoing surveillance [1,4]. Ultimately, the choice between surgical and endovascular strategies and operative strategy should be individualized, taking into account patient anatomy, comorbidities, and institutional expertise [8].

In terms of follow-up of this particular cohort of patients, the AHA/ACC guidelines and recent reviews emphasize the importance of long-term imaging follow-up-preferably using magnetic resonance imaging (MRI) or CT-to detect residual narrowing, aneurysm formation, or pseudoaneurysm [5]. Our case demonstrates an unusual anatomical configuration: a post-ductal coarctation combined with an aberrant right subclavian artery arising immediately distal to the narrowed segment. Conventional resection and interposition grafting were not feasible, as trial clamping of the coarctation and aberrant subclavian artery caused an unacceptable fall in distal aortic pressure. The surgical team, therefore, opted for an extra-anatomic bypass using a Dacron tube graft, effectively excluding the diseased segment while preserving distal perfusion.

At the time of surgery (1989), bypass grafting was less commonly performed compared to resection and graft interposition. However, in this context, the choice was dictated by the patient’s complex vascular anatomy. The result has proven durable, with a competent graft and no re-coarctation 35 years later. This long-term success highlights bypass grafting as a valuable technique in the armamentarium of the adult cardiac surgeon, especially when standard approaches are precluded by unusual anatomy or hemodynamic instability.

## Conclusions

This case highlights the importance of tailoring the surgical management of CoA to the individual patient’s anatomy and intraoperative hemodynamics. While resection with interposition grafting is the standard open repair in adults, bypass grafting offers a safe and durable alternative when conventional repair is not feasible, as in the presence of anomalous branch vessels or intolerance to cross-clamping. The excellent 35-year outcome in this patient underscores the long-term durability of bypass grafting and reinforces its value as an important technique in the surgical armamentarium for complex adult coarctation.
